# Artificial Intelligence in Inflammatory Bowel Disease Endoscopy: Implications for Clinical Trials

**DOI:** 10.1093/ecco-jcc/jjad029

**Published:** 2023-02-22

**Authors:** Harris A Ahmad, James E East, Remo Panaccione, Simon Travis, James B Canavan, Keith Usiskin, Michael F Byrne

**Affiliations:** Bristol Myers Squibb, Princeton, NJ, USA; Translational Gastroenterology Unit, Oxford NIHR Biomedical Research Centre, University of Oxford, Oxford, UK; Inflammatory Bowel Disease Clinic, University of Calgary, Calgary, AB, Canada; Translational Gastroenterology Unit, Oxford NIHR Biomedical Research Centre, University of Oxford, Oxford, UK; Bristol Myers Squibb, Princeton, NJ, USA; Bristol Myers Squibb, Princeton, NJ, USA; University of British Columbia, Division of Gastroenterology, Department of Medicine, Vancouver, BC, Canada; Satisfai Health, Vancouver, BC, Canada

**Keywords:** Endoscopy, imaging

## Abstract

Artificial intelligence shows promise for clinical research in inflammatory bowel disease endoscopy. Accurate assessment of endoscopic activity is important in clinical practice and inflammatory bowel disease clinical trials. Emerging artificial intelligence technologies can increase efficiency and accuracy of assessing the baseline endoscopic appearance in patients with inflammatory bowel disease and the impact that therapeutic interventions may have on mucosal healing in both of these contexts. In this review, state-of-the-art endoscopic assessment of mucosal disease activity in inflammatory bowel disease clinical trials is described, covering the potential for artificial intelligence to transform the current paradigm, its limitations, and suggested next steps. Site-based artificial intelligence quality evaluation and inclusion of patients in clinical trials without the need for a central reader is proposed; for following patient progress, a second reading using AI alongside a central reader with expedited reading is proposed. Artificial intelligence will support precision endoscopy in inflammatory bowel disease and is on the threshold of advancing inflammatory bowel disease clinical trial recruitment.

## Graphical Abstract

**Graphical Abstract:**
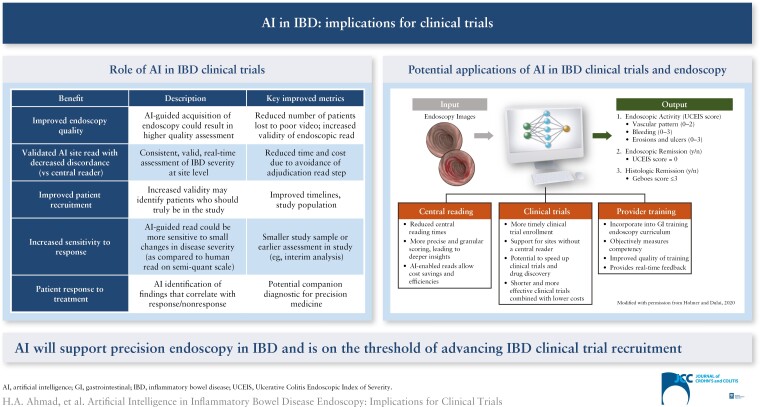

## 1. Introduction

Artificial intelligence [AI] refers to computer algorithms capable of learning, problem solving, and decision making [Table 1].^1–3^ Machine learning is a subfield of AI where algorithms are trained to perform tasks by recognising patterns from data without being explicitly programmed.^4^ Machine learning can evaluate large datasets and detect patterns to assess disease characteristics, such as severity or prognosis.^2^ Clinical applications of AI have expanded across medical fields, including gastroenterology, radiology, pathology, and cardiology.^5^ Importantly, AI is expected to transform endoscopy and image interpretation.^6^

**Table 1.** Artificial intelligence terminology^1–3^

**Artificial intelligence**	The field of computer science which concerns the theory and development of computers to perform tasks that usually require human intelligence, such as image classification, speech recognition, and decision making
**Machine learning**	A field of artificial intelligence that refers to the computers’ ability to learn to make decisions or detect patterns [without explicitly being programmed] from data
**Deep learning**	Subfield of machine learning that exploits many layers of nonlinear information processing for supervised or unsupervised feature extraction and transformation, and for pattern analysis and classification using various neural network frameworks
**Neural networks**	Model of layers consisting of connected nodes broadly similar to neurons in a biological nervous system
**Convolutional neural networks**	Deep learning architecture that adaptively learns hierarchies of features through back-propagation and is used for detection and recognition tasks in images [eg, face recognition]
**Computer-aided detection/diagnosis**	Describes use of a computer algorithm to provide detection [CADe] or a diagnosis [CADx] of a specified object/region of interest
**Supervised learning**	The task of an algorithm learning a function that maps an input to an output based on provided example data
**Unsupervised learning**	The task of a machine learning algorithm to learn the underlying data structure of unlabelled example data—for example, finding commonalities—leading to insights and therefore a greater understanding of the example data
**Classification**	The process of predicting a class/subcategory of given data points from known example data
**Support vector machine**	A discriminative classifier that determines classes from a separating hyperplane; through the use of a kernel, support vector machines can be adapted to suit nonlinear problems

Adapted from Seyed Tabib *et al*. [2020]^2^ and Pannala *et al.* [2020].^3^

Deep learning, a subset of machine learning, uses multilayered artificial neural networks to mimic the human brain and includes convolutional neural networks [CNNs] which are widely used in image and pattern recognition.^5,7^ Network interconnections allow algorithms to optimise classification during training by determining weights and adjusting for factors such as inherent biases or diversity.^1^ Several AI algorithms have been applied to gastroenterology to support computer vision techniques, such as computer-aided detection [CADe] and computer-aided diagnosis [CADx].^6,8,9^ Machine learning algorithms have particular potential for scoring disease activity, refining endpoints, and recruiting patients for trials in inflammatory bowel disease [IBD].^10-13^ Current AI algorithms developed for IBD assessment, and their benefits and limitations in clinical trials, can be found in Table 2.^14–17^

**Table 2.** Examples of current AI algorithms for IBD assessment in clinical trials^14–17^

AI algorithm	Benefits	Limitations
Bayesian additive regression trees	Can establish cause-effect relationship	May not accurately represent the true data generating distribution and therefore may misrepresent the relationship between variables
Gradient boosting machine	Can capture complex relationships between variables to predict events	Clinicians likely not familiar with this methodology
Clustering	Can discover patterns and structure in labelled and unlabelled datasets; unsupervised model	Clustering of clinical data can be hindered by missing variables; can be difficult to cluster multivariate and relatively short time series
Decision tree	Can classify treatment response and predict outcomes	Simplification errors may occur when measuring the benefit of treatment decisions on outcomes such as quality-adjusted life-years; performing a time-consuming analysis adequately in a busy clinical environment may be difficult; various factors in decision making cannot be accurately reflected in a decision tree
Neural network	Can help predict clinical outcomes or make a diagnosis	Difficult to interpret
Random forest	Can predict survival outcome	Not suitable to predict benefit for a specific treatment
Regression trees	Can define prognostic groups for patients due to simplicity and intuitive interpretation	Intrinsic limitations in predictive performance
Support vector machine	Can classify and predict high-dimensional data, including diagnosis, disease course, disease severity, disease subtypes, and medication adherence	Eliminates factors/parameters based on conditional relevance

AI, artificial intelligence; IBD inflammatory bowel disease.

## 2. Current Status of Endoscopy in IBD Clinical Trials

Endoscopic mucosal healing is a therapeutic target for IBD^18^ since it is associated with lower rates of corticosteroid dependency, hospitalisation, and surgery^19^; however, endoscopy has inherent limitations in clinical trials [Table 3].^10,20–23^

**Table 3.** Challenges of IBD endoscopy reading in IBD studies^10,20–23^

Challenge	Description	Implication
Limited local reader expertise	IBD endoscopy evaluation of disease severity varies greatly in expertise across global sites	Improper eligibility/efficacy read, central reader discordance, adjudication reading
Inconsistencies across local reads	Local reads can vary in assessment consistency even within the same site and patient examination	Improper eligibility/efficacy read, central reader discordance, adjudication reading
Poor endoscopy quality	Endoscopies can vary greatly in quality across global sites	Not readable endoscopy assessment, lost time in screening, excluded patient
Local vs central read discordance	Discordance on reads leads to greater costs, long turnaround times, delayed reads	Patient lost to being out of screening window, lost study budget

IBD, inflammatory bowel disease.

### 2.1. Patient recruitment

Endoscopic assessment is central for clinical trial patient selection, including enrolment, stratification, re-randomisation, and open-label drug eligibility.^10^ However, patient recruitment is a significant challenge in IBD clinical trials, partly because physicians are focused on procedures rather than recruitment.^24^ Interobserver variability and endoscopist inexperience may also lead to misevaluation of disease severity, resulting in inappropriate patient enrolment or incorrect treatment arm assignment.^20^ Recruitment inefficiencies may result in the loss of participants from a relatively small pool of eligible patients, creating the need for larger cohorts and increasing clinical trial costs.^10,21,22^

### 2.2. Local versus central reading

To overcome the subjective variability in endoscopic scoring, central reading of endoscopic videos has become commonplace in IBD clinical trials and has been extended to interpretation of histopathological samples.

Local readers tend to overscore the screening endoscopy and underscore the outcome endoscopy. A clinical trial in patients with ulcerative colitis [UC] found that data from local readers supported a marginal difference [30.0% vs 20.6%; *p* = 0.069] in clinical remission between mesalazine compared with placebo.^20^ However, when endoscopic images were reviewed by a single central reader, remission rates were 29.0% versus 13.8% [*p* = 0.011] for mesalazine and placebo, respectively.^20^ Independent assessment excluded 31% of enrolled patients who did not have sufficient endoscopic disease, highlighting the objectivity introduced by central review.^20^ Similar trends in the objectivity of local readers relative to central readers have been reported in a clinical trial in patients with Crohn’s disease [CD].^25^

### 2.3. Endoscopy acquisition and quality

Although central reading decreases interobserver variability and adjudication mitigates subjectivity, these steps are costly.^10^ Machine learning might replace one, two, or all human central readers, resulting in decreased costs and more accurate and consistent reporting.^10^ Additionally, central reading incurs a delay [typically 2 to 3 days], which could invalidate a patient’s eligibility.^26^ The video is sent to a central laboratory for quality control; it is then edited and uploaded to the central reader, who assesses it [usually within 24 h] and returns the reading. Immediate, objective assessments would decrease the delay of central reading.^26^

Central reading is a step forward, but is not the answer to improving the quality of the image and/or data capture. Technique, false interpretations, variability among readers, and missing data contribute to endoscopy quality.^23^ Moreover, inadequate bowel preparation leaving debris that obscures video quality, and endoscope slipping [cinematography] causing blind spots, affect acquisition and quality.^12,23^ Imaging artefacts created by motion, bright pixel areas due to specularity or pixel saturation, or underexposure can limit assessment of underlying tissue.^27^ More than 60% of an endoscopy video frame and nearly 70% of an endoscopy video sequence can be corrupted by artefacts.^28^ An ideal model would be a system that improves the quality of data capture, which in turn improves the performance of endoscopy and provides site-level reading.

### 2.4. Assessment and endpoints

Endoscopic remission in CD and UC and histological remission in UC correlate with improved outcomes in IBD, and both are primary or key secondary endpoints in clinical trials.^19^ Human evaluation of colonoscopy and biopsy interpretation is, however, subjective.^11,29,30^

Scoring systems attempt to provide consistency but were developed using older technologies, often without item-response theory, and are subject to performance limitations.^8,23^ The first endoscopic scores were designed to assess severity rather than extent of endoscopic activity in UC. The Baron Score uses a four-point scale based on severity of mucosal friability and bleeding.^31^ The Modified Mayo Endoscopic Score [MMES] is also used for UC but combines severity of the Mayo Endoscopic Subscore [MES] with extent of disease.^32^ The Ulcerative Colitis Endoscopic Index of Severity [UCEIS] is a validated system used to score vascular pattern, bleeding, and erosions/ulcers in the worst affected area.^33,34^ Unlike the UCEIS, the MES levels overlap, using descriptive terms that are not mutually exclusive, and neither index scores disease extent.

The Crohn’s Disease Endoscopic Index of Severity [CDEIS] assesses ulceration on colonic segments and stenosis using a score from 0 to 44, with higher scores indicating increased severity.^35^ The Simple Endoscopic Score for Crohn’s Disease [SES-CD] evaluates four endoscopic variables [presence and size of ulcers, proportion of surface covered by ulcers, proportion of surface affected by disease, and severity of stenosis] in each of the five ileocolonic segments.^36^ Endoscopists score the variables on a scale of 0 to 3.

Several endoscopic scoring systems have been validated in clinical studies, including the Modified Multiplier Simple Endoscopic Score for Crohn’s disease [MM-SES-CD], Rutgeerts score in CD, and Paddington International virtual ChromoendoScopy ScOre [PICaSSO] in UC. The MM-SES-CD assesses endoscopic severity to predict 1-year endoscopic remission in patients with CD who are on active therapy.^37^ A specialised CD scoring system, the Rutgeerts score, is used for predicting recurrence of disease in patients undergoing ileo-colonic resection.^38^ In a recent post hoc analysis, MM-SES-CD had similar performance to the Rutgeerts score for predicting subsequent clinical recurrence of postoperative CD.^39^ In UC, the PICaSSO score used virtual electronic chromoendoscopy to assess vascular and mucosal features of healing and demonstrated the highest correlation with histology compared with the MES and UCEIS.^40^

The reliability of scoring instruments is measured by intraclass correlation coefficients [ICCs] where: 1 is perfect reliability; 0.9 to <1 indicates excellent reliability; 0.75 to 0.9 indicates good reliability; 0.5 to 0.75 indicates moderate reliability; and <0.5 indicates poor reliability.^41^ The interrater ICC for SES-CD, for example, lies between 0.6 and 0.8 and is heavily dependent upon the level of training [ICC 0.68 for untrained vs 0.93 for trained physicians].^42^

Scoring systems are limited by reader subjectivity and incomplete visualisation of the mucosa, along with inadequate validation and complexity of the scoring instrument.^23,43^ AI can provide objective and consistent assessment of mucosal disease activity, translating into more accurate clinical trial data.

## 3. The Role of AI in IBD Clinical Trials

AI can improve patient recruitment, enhance endoscopy quality, provide a validated site read, increase sensitivity to response, and improve patient treatment response [Table 4].^4,10–13,23,44–51^ Advances in predictive modelling are expected to improve decision making in clinical research programmes and to streamline drug development pathways.^45^ Machine learning techniques have been adopted in trial design to ensure consistent and objective assessment, including patient recruitment.

**Table 4.** Potential benefits of AI application in IBD trials^4,10–13,23,44–51^

Benefit	Description	Key improved metrics
Improved endoscopy quality	AI-guided acquisition of endoscopy could result in higher quality assessment	Reduced number of patients lost to poor video; increased validity of endoscopic read
Validated AI site read with decreased discordance [vs central reader]	Consistent, valid, real-time assessment of IBD disease severity at site level	Reduced time and cost due to avoidance of adjudication read step
Improved patient recruitment	Increased validity may identify patients who should truly be in the study	Improved timelines, study population
Increased sensitivity to response	AI-guided read could be more sensitive to small changes in disease severity [as compared with human read on semi-quantative scale]	Smaller study sample or earlier assessment in study [eg, interim analysis]
Patient response to treatment	AI identification of findings that correlate with response/nonresponse	Potential companion diagnostic for precision medicine

AI, artificial intelligence; IBD, inflammatory bowel disease.

### 3.1. Patient recruitment

AI can help identify appropriate candidates for trial enrolment by matching electronic medical record [EMR] information and other patient data against selection criteria.^4,44,45^ Machine learning algorithms can make a real-time enrolment decision at the site level, with concordance similar to central reading.^10,23,46^ AI could identify patients meeting selection criteria during routine endoscopy who might otherwise not be captured, assuming that consent enables the use of the recorded video. Enhancement of patient cohort selection through AI can increase recruitment efficiency with a smaller, less heterogeneous sample size.^4,10^ In addition, predicting patient response to placebo could lead to increased confidence in patient selection decisions, with the potential for a synthetic control arm.^21,52^ With more reliable prediction of patient outcomes, AI could also support early ‘go/no-go’ decisions for drug development.

### 3.2. Rapid endoscopic results

Additional tools to expedite central reading are needed to reduce delays in clinical trials.^10,11^ AI-assisted assessment of disease activity is expected to decrease variability and minimise the need for second reader/adjudication.^23^ With AI, endoscopic assessment would instantly be available at the local site to provide a score upon the site’s submission to the central laboratory. This would eliminate the central reading delay, which would be consigned to ‘over-reading’ rather than primary reading.

### 3.3. Cost savings

The estimated average cost for IBD clinical trials ranges from $30 million for a pivotal clinical trial to $55 million [US dollars] for phase I through phase IV trials.^53,54^ AI has the potential to reduce central reading cost, which accounts for a considerable portion of trial budgets. AI can reduce the cost of video equipment and digitalisation of histology slides necessary to perform offsite analysis.^26^ One study estimated that AI-assisted optical biopsy for colon polyps would decrease the costs of colonoscopy by 10.9% or by $85.2 million per year in the USA alone.^55^ In the absence of a cost savings value in IBD, the savings seen for colon polyps may provide a perspective.

### 3.4. Improving endoscopic assessment with AI

**Figure 1.**
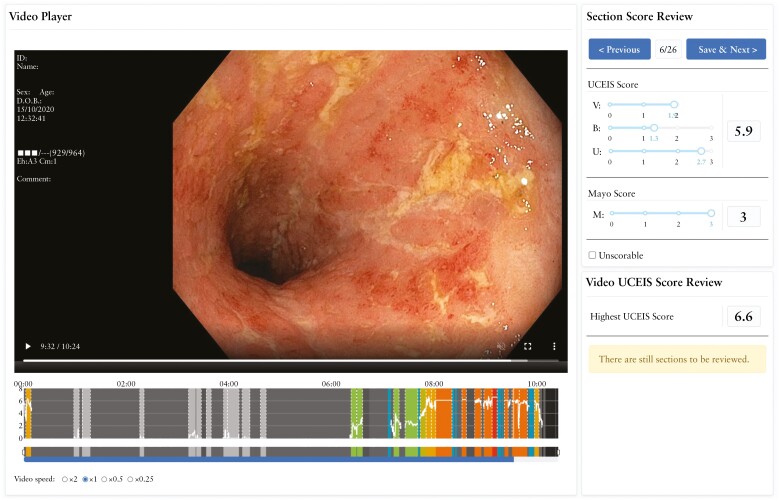
Example of AI-assisted endoscopy interface. Image provided by Dr Michael Byrne on behalf of Satisfai. AI, artificial intelligence; B, bleeding; U, ulceration; UCEIS, Ulcerative Colitis Endoscopic Index of Severity; V, vascular pattern.

Clinical trials have successfully used AI in IBD endoscopy, including CADe [eg, polyp detection], CADx [eg, polyp classification], and improvement [eg, scoring bowel preparation], demonstrating its ability to advance endoscopic quality while decreasing interobserver variability.^12,47–51^ An example of an AI-assisted endoscopy interface can be found in Figure 1. AI can outperform humans, does not get tired or impatient, and does not have a limited attention span; therefore, it is less likely to miss subtleties.^2,8,10,13^

Endoscopic techniques for polyp detection and assessment of IBD differ. This matters for both CD and UC because biopsy bleeding, friability, or scope trauma may be scored as a consequence of disease activity. AI algorithms can be trained to differentiate this type of bleeding from disease severity.

Examples of AI with the potential to improve endoscopic assessment of disease include Red Density, EndoBRAIN, and PICaSSO. An operator-independent, computer-based tool, Red Density can score disease activity in UC using a redness map and vascular pattern recognition.^56^ This score had significant correlation with the histological scoring systems [Robarts histopathology index] and with MES and UCEIS endoscopic scores. Due to its high level of performance and algorithm structure, Red Density does not require as much information as the CNN and presents an important application of AI. Another example where AI has improved the assessment ability of endoscopy is the EndoBRAIN system, which has demonstrated the ability to detect high-grade dysplasia in patients with long-standing UC who subsequently underwent an endoscopic submucosal dissection.^56^ Because diagnosis of colitis-associated colorectal cancer may be difficult due to inflammation-associated consequences on mucosal appearance, the use of EndoBRAIN could help less experienced endoscopists with identification of lesions. The PICaSSO is the first validated endoscopic score using the new generation of virtual chromoendoscopy endoscopes in UC. This score had a very good interobserver agreement in the pre-test and post-test evaluations that could reflect the full spectrum of mucosal and vascular changes, including mucosal healing in UC.^57^

### 3.5. Quality of examination metrics

Automated quality of examination [QoE] metrics can improve endoscopy examination and provide real-time feedback.^58^ For example, AI can alert the endoscopist if the withdrawal time [a quality metric for polyp detection] is below a predefined threshold.^12,59,60^ Meta-analysis of prospective trials found that AI-based polyp detection systems increased the detection of non-advanced adenomas and polyps, compared with standard colonoscopy.^60^ Since IBD clinical trials exclude patients with neoplasia, AI could be useful for excluding ineligible patients.^61^

QoE metrics can be incorporated into machine learning algorithms designed to prevent the collection of poor-quality videos, by alerting the user and reducing the need for a patient to return for re-evaluation. For quality assurance, AI can report on the total percentage of colonic surface area visualised, bowel preparation, and resolution of the endoscopic image.^12,58,62^ This facilitates a thorough examination, which is in everyone’s interests, including the patient’s.

By way of example, AI helps real-time differentiation of adenomas from post-inflammatory polyps.^9^ A deep CNN applied to 125 consecutive colonoscopy videos was able to differentiate between hyperplastic polyps and adenomatous polyps, with an accuracy of 94%, a sensitivity of 98% [95% CI 92%–100%], and specificity of 83% [95% CI 67%–93%].

A real-time quality improvement system [WISENSE] was developed to monitor blind spots, record procedure time, and generate photodocumentation during 324 consecutive oesoph-agogastroduodenoscopies.^48^ WISENSE had a 90% accuracy for monitoring blind spots and significantly decreased the rate of blind spots compared with the control group [5.9% in WISENSE group vs 22.5% in control group; *p* <0.001]. A deep learning model has been shown to assess missed areas during colonoscopy, using depth and pose estimation, providing segment by segment coverage with 93% agreeing with the physician reviewer.^62^

AI can restore corrupted data in endoscopic imaging. Using a dataset of 1290 endoscopy images, a CNN detector processed artefacts with indefinable shapes and generated a quality score for each video frame. The model had a mean average precision [mAP at 5% threshold] of up to 49.0 and a computational time as low as 88 ms, allowing real-time processing. The detector was also able to restore approximately 25% of the video frames to increase the overall frame retention rate to nearly 70%.^27^

The European Society of Gastrointestinal Endoscopy recently developed the key performance indicators [KPIs] that should be part of and adopted in every IBD endoscopy unit.^63^ Important KPIs are bowel preparation, photodocumentation, number of biopsies, standardised endoscopic scores, and detection rate of dysplasia associated with IBD. These quality metrics should also be incorporated in future clinical trials. AI may play a role in automating KPI metrics and improve the quality and ability of clinical trials to meet their objective.

### 3.6. Assessment of clinical trial endpoints

Image-based endpoint detection using machine learning capabilities has led to more reliable and efficient endpoint assessment.^4^ Deep learning algorithms can analyse large volumes of imaging data, enabling objective evaluation of endoscopy.^2^

Current scoring systems are limited by design—the UCEIS evaluates the worst segment of the lesion as opposed to integrating multiple areas, and SES-CD is affected by significant subjectivity.^23^ Computer vision algorithms can provide cumulative quantification of erosions/ulcers, of the affected area or normal mucosa, or of endoscopy quality.^2,11,64^ In an analysis of the mirikizumab phase 2 trial, the ability of a recurrent neural network [RNN] to predict central reader scores was compared with the UCEIS and MES scoring systems.^10^ A total of 795 full-length endoscopy videos from 249 patients was analysed by central readers and used to train the RNN. The study showed excellent agreement with human central reading scores, with an endoscopic healing accuracy of 97.0% and 95.5% for the UCEIS and MES, respectively.^10^

Recognising the comprehensive assessment by AI algorithms, some models exploit spectral characteristics and tissue colour to detect inflammation over a larger area compared with conventional scoring systems. For example, a model was trained to differentiate epithelial tissue of IBD and control patients from other tissue, as a first step, using Raman spectra as a second step to classify the sample as CD, UC, or healthy.^65^ In a cross-sectional analysis of 38 patients [14 patients with CD, 13 patients with UC, and 11 healthy controls], Raman spectroscopy classified each group with 98.9% accuracy, 99.1% sensitivity, and 98.1% specificity for detecting healthy controls.^65^ Furthermore, a trained neural network using Raman spectroscopy has been developed that can accurately differentiate mucosal healing from active inflammation in CD and UC.^66^

Although AI can already match image interpretation by experienced gastroenterologists, the aim should be to exceed the abilities of skilled physicians.^13^ A real-time, operator-independent tool based on Red Density can now accurately identify inflammation and assess UC disease activity.^11^ Red Density uses an algorithm built from 29 patients with UC and six healthy controls, based on the red channel of the red-green–blue pixel values and pattern recognition. The Red Density score significantly correlated with the Robarts histological index [*r* = 0.65, *p* <0.0001], MES [*r* = 0.61, *p* <0.0001], and UCEIS [*r* = 0.56, *p* <0.001].^11^ Another study used 8000 images to train and validate three different CNN models. The models distinguished eight classes of anatomical gastrointestinal landmarks and diseases, with accuracies approaching 99%.^67^

Using improved endoscopic assessment tools to predict long-term clinical outcomes is a critically important role of AI in IBD. A study by Maeda and colleagues was the first that analysed the relationship between real-time AI-assisted colonoscopy outputs and the long-term prognosis of patients with UC. The findings showed that this fully automated AI system was able to assess the risk of clinical relapse in patients with UC in clinical remission, therefore enabling the clinicians to make real-time treatment decisions.^68^

AI has the potential to become the gold standard for assessing disease severity.^8^ Systems are being developed to standardise scoring of difficult parameters, such as endoscopic healing.^64^ AI-derived endoscopic assessment in clinical trials can be expected to lead to predictive scoring measures and to evolve into a machine that produces scores for the endoscopist related to outcomes, which may reduce the heterogeneity of treatment decisions.

### 3.7. Defining remission

Remission matters, and an accurate definition is an extension of improved disease activity scoring. One deep learning algorithm used a CNN-graded endoscopic severity rating in 3082 patients with UC to discriminate between disease remission [MES 0 or 1] and moderate to severe disease activity [MES 2 or 3].^13^ Weighted kappa scores showed almost perfect agreement between the deep learning model and human reviewers in grading endoscopic severity [0.86, 95% CI 0.85–0.87].^13^ A study of 841 patients with UC was able to identify MES scores of 0 and 0 to 1 with area under the receiver operating characteristic curves [AUROCs] of 0.86 and 0.98, respectively, using a CNN-based CAD system for endoscopic severity.^69^ Notably, the CNN performed better in the rectum than in the right and left colon for an MES score of 0 [AUROCs = 0.92, 0.83, and 0.83, respectively].^69^ Using data from a single-centre retrospective cohort, a machine learning algorithm predicted remission using laboratory values and patient age in 1080 patients receiving thiopurine therapy.^70^ The five most important predictor variables included haemoglobin, lymphocytes, haematocrit, neutrophils, and platelets. The algorithm differentiated remission from non-remission in the validation dataset, with an AUROC of 0.79, versus 0.49 using 6-thioguanine nucleotide metabolite levels.

Beyond the human eye, AI can explore new definitions of remission, such as quantifying vascular pattern, light reflex, or the pallor of normal mucosa. AI can also assist real-time histological evaluation. A deep neural network based on endoscopic images of UC [DNUC] was developed to predict histological remission.^46^ For endoscopic remission, the DNUC was sensitive [93.3%] and specific [87.8%], with a diagnostic accuracy of 90.1%. For histological remission, the DNUC demonstrated 92.4% sensitivity, 93.5% specificity, and 92.9% diagnostic accuracy.^46^ Since histological remission is associated with a better long-term outcome, detection in real time by the DNUC has immediate implications for clinical trials and practice.^19^

### 3.8. Integration of data

The potential to assimilate data sources from IBD datasets [including clinical symptoms, endoscopic read-outs, histopathology, gene expression values, and other outcomes] represents multiparametric data analysis that can provide further insight from clinical trials.^10,45,52,71^ Analysis of large genomic, transcriptomic, proteomic, and microbiomic [multiomic] datasets by machine learning could lead to the discovery of novel, clinically relevant biomarkers.^72^ Enormous opportunities to transform the IBD field lie at the intersection of multiomics, pathology, and endoscopy with AI solutions.^73^

Indeed, multiomics potentially predicts IBD treatment outcomes.^74,75^ In the precision medicine era, AI could provide detailed insight into a patient’s molecular profile and inform prognosis, disease aetiology, and/or therapeutic response.^2,74^ Faced with the challenge of unevenly sampled and sparse clinical time series data, a novel approach founded in extreme value theory [EVT] was deployed to convert these measurements into interpretable metrics of patient abnormality. Machine learning techniques and EVT methods were able to compare adalimumab and infliximab over several years in terms of relative effectiveness, predicting patient response and characterization of this response.^76^

### 3.9. Training opportunities

AI can support the education and training of endoscopists. Experienced endoscopists already gain valuable knowledge from the feedback of central readers. Real-time feedback delivered by AI can serve as an extension of training and can bolster examination quality by guiding endoscopists as they perform the procedure, assisted by an accurate tool that can provide on-the-job education and constructive feedback.^26^

## 4. Limitations of AI in Clinical Trials

Datasets are selected and categorised by humans, which may bias AI algorithms.^26^ Model accuracy depends on the degree to which endoscopists correctly provide scoring information.^7^ Current methods use supervised learning during which the algorithm is trained to make the same decision as physicians. Using unsupervised learning would identify clinically relevant patterns within data without ground truth information, which may be an effective strategy to avoid bias.^7^ Other approaches to improve ground truth measurements include agreement with a central reader IBD group using a Delphi procedure, correlation with histology/transcriptomic data, and correlation with longer-term clinical outcomes as ground truth. AI should decrease intra-observer variability and the assumption might be that the kappa for intra-observer variation in AI is 1.00, but this has yet to be examined and will be a determinant of the ability of AI to reduce variability. Models need to be trained on the differences between CD and UC, where the latter assessment of endoscopic findings and histology from mucosal biopsies might give a less complete picture [and ability to prognosticate outcomes] than the former, because of the transmural process. Additionally, AI models will need to be trained to ignore biopsy bleeding/friability; this is particularly true for UC assessment, because mucosal bleeding needs to be assessed ahead of the scope. That includes education of endoscopists regarding technique, to optimise views during scope insertion, in contrast to spotting polyps on withdrawal.

Endoscopic interpretation is potentially biased by clinical information, but that depends on the clinical context [discriminating ischaemic from UC, for example], although some scoring systems are not biased by clinical information.^20,77^ Rare clinical scenarios also challenge AI systems, since they have less representation within training datasets. An example is distinguishing Behcet’s [more common in East Asia] from CD. Thus, high-quality datasets are needed to ensure geographical, technical, and patient demographic diversity.^1^ The American Society for Gastrointestinal Endoscopy proposes a professionally managed image library,^6^ but the requirements to ensure correct diagnosis or ground truth for publicly available datasets are not always clear. Some datasets are clearly annotated [eg, the SUN Colonoscopy Video Database^78^].

Pharmaceutical groups, by the nature of regulatory requirements, are likely to hold high-quality datasets that are specific to IBD trial populations and would be optimal for AI development. Collaboration with pharmaceutical groups [eg, endoscopic video resource from their anonymised trial videos] would complement the work of the Foundation for the National Institutes of Health [FNIH] Biomarkers Consortium on mucosal healing [sponsored by contributing Pharma [Bristol Myers Squibb, Lilly, Johnson & Johnson, Takeda]]. The large number of trial patients in endoscopic remission would provide potential videos and linked histology to assist in a deep dive into the definition of remission. EMR systems would provide a useful resource for algorithm development.^79^

Standardisation of image capture is necessary to train algorithms to reflect clinical scenarios.^6,26^ Examination quality can be determined by automated analysis of endoscopy videos, facilitating the exclusion of poor-quality data.

The use of video capsule technology has been evolving in the field of CD. It has been applied to diagnosis and assessment of mucosal healing in the small bowel. However, limitations of this technology include the large amounts of data collected and consequent long duration of the analysis, both of which may be overcome with AI.^56^ AI could enable selection of the frame or the section of video needed for the assessment, shortening the time for diagnosis and requiring a limited amount of data storage. However, obstacles remain in the development of AI for video capsule endoscopy [CE] that must be overcome in order for this technology to be implemented in the clinic. Current obstacles include: [a] use of retrospective data from single centres or small patient cohorts that restrict the generalisation of the established CNN systems and lead to lack of validation of the AI system; [b] use of single images, not the entire video, so that the analysis is not able to provide an overall evaluation of the validated scores for video capsule [eg, the Lewis score]; [c] uncertain performance of the CE in real-world practice due to potentially low quality of CE images; and [d] lack of use of CE data from various kinds of CE systems and diverse clinical situations.^80^ Despite enthusiasm, problems with implementing AI in CD assessment remain and can be attributed to lack of education and knowledge among IBD providers, as well as hesitancy related to the potential of AI to replicate or replace expert clinical judgement.

Whereas early attempts to investigate AI for diagnosing dysplasia and identifying neoplastic lesions in IBD show promise,^56^ the need for human assessment is likely to remain in the immediate future, even as algorithms advance in the use of inflammation assessment. Development of an AI algorithm for digital pathology that is capable of recognising and characterising dysplasia in IBD remains a challenge, as further improvements in diagnostic performance are needed.

Technological risks are inherent to AI due to the large amounts of data involved. Considerations regarding the nature of the data, patient privacy, cyber security, and potential roles of these algorithms are all of paramount importance in AI design. Many AI systems run as updatable software on a hardware platform. Updates are likely to be downloaded from the internet, making AI systems hackable or allowing unscrupulous actors to introduce errors that may reduce performance or even install ransomware. Commercial, malicious, or fictitious attacks on AI may invalidate a trial even if there are no patient safety risks.^81^ These are material concerns. Some facilities have technical limitations that present barriers to using AI tools [eg, hospital Wi-Fi networks may limit the ability to upload and assimilate data, although 5G may provide adequate bandwidth].

Clinical trial inclusion and exclusion criteria present barriers to enrolment. Sadly, some investigators may be tempted by financial conflicts of interest to override trial entry barriers for monetary gain.^82^ Central reading mitigates this possibility and AI may prevent such non-adherence. AI already supports existing models that use data to identify non-adherence and inappropriate subject enrolment.^82^

IBD clinical trials typically cost between $30 and $55 million [US dollars],^53,54^ with a considerable portion of trial budgets spent on central reading costs. Whereas there is potential for AI to decrease costs of clinical trials in IBD, particularly with regard to central reading, the true cost savings remain unclear. The operational challenges of integrating AI technologies into existing setups may be a burden on sites that require additional hardware, personnel, or time. In addition, there has not yet been a prospective, multicentre, clinical trial in IBD where the sponsor implemented AI reading at trial initiation. All studies using AI in IBD have been post hoc evaluations or single-centre, prospective studies. Full incorporation of AI in a global clinical trial will be an important step in promoting widespread use.

## 5. Regulatory Opportunities

FDA guidance for industry on UC clinical trial endpoints recommends that endoscopic assessment is made by both the endoscopist and the blinded central reader.^83^ The FDA also recommends that the study protocol specifies how discrepancies between the assessments of the endoscopist and the central reader will be handled in the efficacy analysis. Charters that standardise the methodology underlying the scoring of endoscopic characteristics that may have subjective elements are particularly important. The FDA recommends the involvement of central reading for histological evaluations of biopsy specimens, including charters that standardise procedures and assessments. Machine learning techniques can increase consistency, objectivity, and accuracy of assessments, so their incorporation could help meet FDA recommendations.

The FDA regulatory framework distinguishes between technologies that ‘drive’ or that ‘inform’ clinical management.^84,85^ Within this framework, CADe and CADx are positioned as technologies to drive clinical management through predicting disease risk or aiding in diagnosis. Many AI applications have already received regulatory approval, including approaches for detecting atrial fibrillation, diagnosing diabetic retinopathy, interpreting magnetic resonance imaging, and diagnosing intracranial haemorrhage.^81^ Technologies with the ability to improve consistency in objective assessment are likely to be well received by regulatory agencies, in the same manner that central reading in IBD clinical trials was adopted because of its impact on consistency.^20^

## 6. Future Directions

In the future, more prospective studies that will allow for better definition of the role of AI, implementation of AI into multicentre, global, prospective, IBD clinical trials, and development of optimal algorithms for both endoscopic and histological assessment of IBD in clinical trials will be needed. Up to now, almost all studies using AI in IBD have been post hoc evaluations or single-centre, prospective studies. One of the few examples of a multicentre, international, prospective study with a large cohort of patients, and with many AI endoscopy videos as well as histological slides, was the study in which PICaSSO endoscopy and histology AI were developed.^40^ The AI was able to predict endoscopic inflammation/remission and long-term clinical outcomes in white-light endoscopy and virtual electronic chromoendoscopy. In a recent publication,^86^ a new UC histological score that can be incorporated into an AI algorithm was developed, the PICaSSO Histologic Remission Index [PHRI]. This AI algorithm based on PHRI differentiated active from quiescent UC with high accuracy, sensitivity, and specificity; it also had the highest correlation with endoscopic activity and clinical outcomes. However, further validation of this approach, as well as the pairing of AI endoscopy with digital pathology in multicentre studies, is needed for the future direction for AI, as these improved assessments will be tied closely to important clinical patient outcomes.

**Figure 2.**
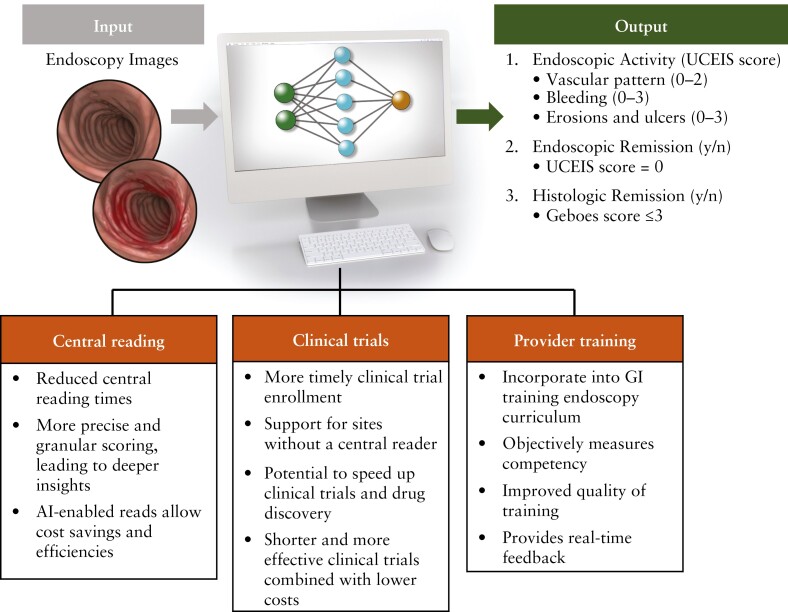
Potential applications of AI in inflammatory bowel disease clinical trials and endoscopy.^26^ AI, artificial intelligence; GI, gastrointestinal; UCEIS, Ulcerative Colitis Endoscopic Index of Severity. Modified with permission from Holmer and Dulai, 2020.^26^

AI has the potential to advance IBD clinical trials and support the quality of IBD endoscopy [Figure 2]. In the near future, regulatory applications will be filed to embed AI into the trial process at all levels, including assessment of primary endpoints [possibly as second or third readings]. By improving the quality of clinical assessment and allowing greater sensitivity between treatment groups, AI has the potential to decrease sample sizes and costs. Combining natural language processing of EMR data or endoscopy request forms will help pre-identify patients suspected of having IBD, with or without inflammation scoring, to facilitate [and possibly automate] trial enrolment. It would be possible to match eligible patients directly to study teams, including those at other sites, to expand the trial site footprint. With its ability to considerably enhance trial efficiency and reduce costs, AI technology is on the cusp of transforming IBD clinical trials.

## Data Availability

No new data were generated or analysed in support of this research.
